# Hierarchical Nanoporous Sn/SnO_x_ Systems Obtained by Anodic Oxidation of Electrochemically Deposited Sn Nanofoams

**DOI:** 10.3390/nano10030410

**Published:** 2020-02-26

**Authors:** Magdalena Gurgul, Anton S. Lytvynenko, Magdalena Jarosz, Karolina Gawlak, Grzegorz D. Sulka, Leszek Zaraska

**Affiliations:** 1 Department of Physical Chemistry and Electrochemistry, Faculty of Chemistry, Jagiellonian University, Gronostajowa 2, 30-387 Krakow, Poland; gurgulm@chemia.uj.edu.pl (M.G.); anton.s.lytvynenko@gmail.com (A.S.L.); jarosz@chemia.uj.edu.pl (M.J.); gawlak@chemia.uj.edu.pl (K.G.); sulka@chemia.uj.edu.pl (G.D.S.); 2 L.V. Pisarzhevskii Institute of Physical Chemistry of the National Academy of Sciences of Ukraine, Prospekt Nauky 31, 03028 Kyiv, Ukraine

**Keywords:** Sn foams, electrodeposition, tin oxides, anodization, nanopores

## Abstract

A simple two-step electrochemical method for the fabrication of a new type of hierarchical Sn/SnO_x_ micro/nanostructures is proposed for the very first time. Firstly, porous metallic Sn foams are grown on Sn foil via hydrogen bubble-assisted electrodeposition from an acidulated tin chloride electrolyte. As-obtained metallic foams consist of randomly distributed dendrites grown uniformly on the entire metal surface. The estimated value of pore diameter near the surface is ~35 µm, while voids with a diameter of ~15 µm appear in a deeper part of the deposit. Secondly, a layer of amorphous nanoporous tin oxide (with a pore diameter of ~60 nm) is generated on the metal surface by its anodic oxidation in an alkaline electrolyte (1 M NaOH) at the potential of 4 V for various durations. It is confirmed that if only optimal conditions are applied, the dendritic morphology of the metal foam does not change significantly, and an open-porous structure is still preserved after anodization. Such kinds of hierarchical nanoporous Sn/SnO_x_ systems are superhydrophilic, contrary to those obtained by thermal oxidation of metal foams which are hydrophobic. Finally, the photoelectrochemical activity of the nanostructured metal/metal oxide electrodes is also presented.

## 1. Introduction

Nanoporous metal foams (metal nanofoams) are a relatively new class of materials intensively investigated by the research community [1,2]. These three-dimensional structures built by interconnected nanoparticles and/or nanosized filaments represent a unique combination of properties typical for nanostructured (e.g., ultralow density, high porosity, and surface area [3,4]) and bulk metals (such as high thermal and electrical conductivity [1,5]).

Among techniques used for the preparation of metal nanofoams, electrodeposition seems to be exceptionally encouraging [4,6,7,8]. The formation of metallic nanofoams by electrodeposition is based on the electrochemical reduction of metal ions at high current densities accompanied by the intensive formation of hydrogen bubbles playing a role of a dynamic template that is responsible for the metal foaming [9]. The approach allows us to obtain deposits with much higher surface area compared to a standard plain foil [10] and, in contrast to other reported methods, does not require either sophisticated equipment or complex procedures. Another attractive feature of this process is the formation of hierarchically organized micro/nanostructures [11] with higher accessibility of the inner surface to external agents compared to bulk particles of nanoporous solids (as additional microstructuring should shorten the length of nanopores thus helping to mitigate possible diffusion limitations within them). Moreover, electrodeposition leads to the formation of nanofoams as integral coatings adhered to the surfaces of bulk supporting electrodes. The materials obtained in such a way are considered as promising for a number of applications, in particular for a broad scope of electrochemical ones [11,12]. Nanofoams of a set of metals (Cu, Ag, Pt, Pd, etc.) [11,12,13,14] and alloys [6] were obtained by electrodeposition, in particular, successful fabrication of Sn nanofoams has been reported [4,8,15].

The search for environmentally-friendly strategies of energy conversion and storage is nowadays one of the most urgent needs of mankind. Thus, the range of materials currently being studied for these applications is still extensively growing. Among them, tin oxides (mainly SnO_2_, but also Sn_3_O_4_ and SnO), attract great scientific attention due to their unique semiconducting, optical, and electronic properties [16,17,18], which make them promising candidates for photoelectrochemical (PEC) [19] and photovoltaic applications [20]. They also have been considered as favorable materials for various energy storage systems, especially Li-ion and Na-ion batteries [21,22]. It is well known that precisely designed nanomorphologies of such oxides can offer some enhanced features (e.g., electron mobility, surface area) that make them even more attractive for the purposes mentioned above [23].

Among diverse methods of synthesis of nanostructured tin oxides, one of the most attractive is electrochemical oxidation (anodization) of Sn because of its simplicity, low-cost, high effectiveness, and possibility of controlling and tuning the morphology of nanostructures [24,25]. Despite extensive studies on the influence of various anodizing conditions (e.g., applied potential, electrolyte composition, process duration) on the growth and morphology of porous anodic SnO_x_ layers, which have already been performed [24,25,26,27,28,29,30,31,32], nanostructured anodic tin oxide films have been obtained mostly on tin foils [24,26,28,30,32] or smooth Sn layers electrochemically deposited on conductive supports [25,27]. To the best of our knowledge, no researches concerning possibilities of fabrication of nanoporous SnO_x_ on micro/nano-structured metallic substrates have been reported, except the approach proposed by Wu et al. [33] based on the oxidation of Sn nanowire arrays prepared using template-assisted electrodeposition. In particular, the formation of porous tin oxide layers by anodization of Sn nanofoams has not been studied so far, while it seems to be especially promising. Such a process is expected to yield hierarchical Sn/SnO_x_ systems consisting of thin films of nanoporous SnO_x_ generated on dendrites with preserved metal cores. On the one hand, the metal core can act as an effective current collector when the Sn/SnO_x_ system is used as a photoelectrode in photoelectrochemical systems [34]. On the other hand, when it is used in Li- or Na-ion batteries, a combination of the micro/nanoporous structure of Sn dendrites and nanoporous morphology of the oxide film not only provides shorter diffusion paths for lithium or sodium ions but also suppresses volume changes occurring during intercalation/deintercalation that results in enhanced integrity of the electrode [35,36].

Another important property of such hierarchical Sn/SnO_x_ systems is their wettability. Cao et al. [37] reported that self-passivated electrochemically deposited nanoporous Sn foams exhibit hydrophobic or even superhydrophobic behavior without any further surface modification. Such kind of conductive superhydrophobic metal surfaces can exhibit enhanced corrosion resistance and offer potential use in, e.g., microfluidic devices, and water-oil separation, etc. [38,39]. Nevertheless, for several applications, including photoelectrochemical water splitting, good wettability of the nanostructured electrode is strongly desirable. Since we recently confirmed the hydrophilic nature of nanoporous SnO_x_ electrochemically grown on the surface of Sn foil [40], it can be expected that the presence of such kind of porous oxide film can strongly affect the wetting behavior of Sn foams. However, the factors which govern the wetting behavior of these materials remain obscure.

Therefore, here we present, for the very first time, an effective strategy for the fabrication of hierarchical Sn/SnO_x_ micro/nanostructures by electrodeposition of Sn foams followed by their one-step anodization in an alkaline electrolyte. We demonstrate that it is possible to obtain crack-free continuous porous oxide layers on a nanoporous metal foam substrate. The wettability of the prepared composites was evaluated and compared with the Sn/SnO_x_ composites obtained via aerial oxidation of the tin nanofoams. Finally, the photoelectrochemical activity of the obtained material is also shown.

## 2. Materials and Methods 

### 2.1. Substrate Preparation

Sn foil (99.99%, Goodfellow Cambridge Ltd., Huntingdon, UK, GB) was cut into coupons (c.a. 1 cm × 2 cm), washed in acetone and ethanol (both Chempur, Piekary Śląskie, Poland) to remove grease and other surface impurities. After that, the samples were treated with 60, 220, and 800 grit sandpaper in order to define a reproducible surface roughness (increased compared to the as-received foil) and cleaned ultrasonically in isopropanol (Chempur, Piekary Śląskie, Poland). Before electrodeposition, the working surface area was activated by immersing in 36–38% HCl (Sigma Aldrich, St. Louis, MO, USA) for 5 min in order to remove the superficial oxide layer that could emerge from the aerial tin oxidation. Finally, the samples were rinsed in isopropanol one more time.

### 2.2. Fabrication of Sn Foams

Tin foams were fabricated by cathodic electrodeposition in a typical two-electrode configuration with a Sn plate and platinum mesh used as cathode and anode, respectively. Electrodeposition was carried out at room temperature under the constant voltage of 6 V provided by a DC power supply (Array 3646A, Array Electronic Co., Ltd., Taiwan) for 60 s in the electrolyte containing 20 mM SnCl_2_·2H_2_O and 1.5 M H_2_SO_4_ (Sigma Aldrich). The distance between electrodes was fixed at 1 cm. After deposition, the samples were carefully rinsed with isopropanol to remove residues of the electrolyte. The working surface area of samples was defined by insulating the part of the surface with paraffin.

### 2.3. Synthesis of SnOx Layers on Sn Foams

The anodization was also carried out at room temperature in a two-electrode system with the prepared tin foams used as anodes and platinum mesh serving as a cathode. The process was performed in 1 M NaOH (Sigma Aldrich) at the constant potential of 4 V for 10, 20, and 30 min. Then, as anodized SnO_x_ foams were rinsed in isopropanol and water, and dried in air. After that, some samples were annealed at 200 °C for 2 h in air with a heating rate of 2 °C min^−1^ using a muffle furnace (FCF 5SHM Z, Czylok, Poland). 

### 2.4. Materials Characterization

The morphology of the materials was verified using an optical microscope (Delta Optical Evolution 100 Trino Plan with attached Delta Optical DLT-Cam PRO 3 MP digital USB camera as well as with an external LED lamp for upper illumination, Delta Optical, Poland) and Field-Emission Scanning Microscope (FE-SEM/EDS, Hitachi S-4700, Japan with Noran System 7). All geometrical parameters, including the thickness of films and pore sizes, were verified right from FE-SEM images using WSxM v.12.0 software [41].

X-ray diffraction (XRD) measurements were performed using the X-ray diffractometer Rigaku Mini Flex II with monochromatic Cu Kα radiation (λ = 1.5418 Å) in the 2θ range of 20°–80° with a scan speed of 0.5° min^−1^ and a step size equaled to 0.02°.

Photoelectrochemical (PEC) measurements were carried out using a photoelectric spectrometer combined with a potentiostat (Instytut Fotonowy, Kraków, Poland) equipped with the 150 V Xe arc lamp. PEC tests were performed in a three-electrode cell with a quartz window. An Sn/SnO_x_ sample serving as a working electrode was illuminated with a monochromatic light in the wavelength range of 250–500 nm. A Pt wire and saturated calomel electrode (SCE) were used as a counter and reference electrodes, respectively. Photocurrents were recorded in a borate buffer solution (pH ≈ 7.5) at the potential of 0.9 V vs. SCE.

The wettability of the tested samples was verified with the means of water contact angle (WCA) measurements using an OCA25 goniometer (Data Physics, San Jose, CA, USA) with an automatic dosing system. On each Sn/SnO_x_ surface, three separate droplets were put, and 2 min-long movies were recorded in order to monitor the changes in the wettability. Where possible, CA was calculated based on the sessile drop method. The average CA is based on ten consecutive measurements on each droplet. All of the measurements were conducted at room temperature and humidity.

## 3. Results

### 3.1. Electrodeposition of Sn Foams

During the metal electrodeposition in strongly acidic electrolytes at high current densities, an intense hydrogen evolution reaction (HER) is typically observed on the electrode surface. This phenomenon is firmly in line with the ragged current density vs. time curve recorded during electrodeposition at the aforementioned conditions (see Figure S1 in the Supplementary Information). As mentioned above, hydrogen bubbles arising from the reduction of H^+^ ions play a role of a “soft template” during the process, and because of their periodic generation and detachment, the metal foam featured with irregularly shaped voids is formed on the conductive substrate (here Sn foil) due to deposition of tin into the gaps between the attached bubbles. Moreover, high potentials and, consequently, current densities favor the intensive metal nucleation [42] followed, in particular for Sn, by the formation of highly dendritic deposits [43], which, acting simultaneously with the above-mentioned effect of bubble template, leads to the generation of the hierarchically structured material.

The procedure of substrate surface pre-treatment was first considered in order to achieve possibly the most stable Sn foams that can be further electrochemically oxidized without being damaged or delaminated from the underlying tin foil. Four different types of substrates were tested: as-received (degreased) Sn foil, Sn foil roughed with 60, 220, and 800 grit sand-paper, Sn foil activated in HCl, and Sn foil, both roughed and activated. The optical microscope images of particular substrates together with Sn foams deposited on them are collected in Figure S2 (see the Supplementary Information). In general, it was impossible to obtain the Sn foam fully covering the substrate when no pre-treatment was applied before the electrodeposition. The resulting deposit only partially covers the Sn foil, and most of the electrodeposited foam delaminated from the surface just during sample removal from the electrolyte due to its extremely poor adhesion. After some preliminary experiments, it was confirmed that adequate preparation of the substrate surface leads to obtaining nanoporous foams, which uniformly covers the entire surface subjected to electrodeposition. For this reason, all foams used in further studies have been electrodeposited on previously pre-textured and activated metal surfaces.

**Figure 1:**
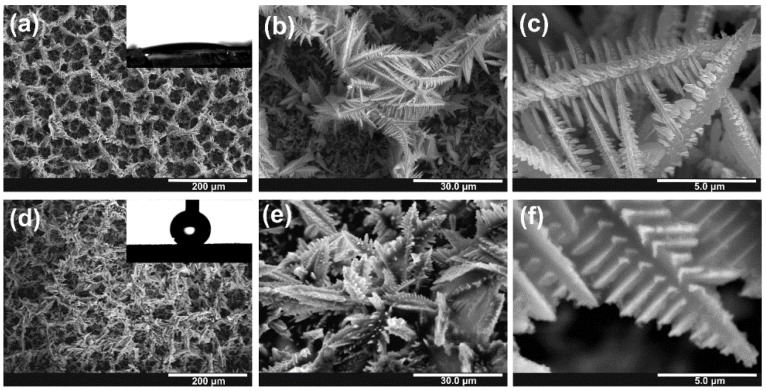
Field-Emission Scanning Microscope (FE-SEM) images of the nanoporous Sn foam obtained by electrodeposition for 60 s in acidulated 20 mM SnCl_2_·2H_2_O at the potential of 6 V. As-obtained (**a**–**c**) and annealed in air at 200 °C for 2 h (**d**–**f**). Insets in **a** and **b**—images of water droplets just after placing on the particular surface.

**Figure 2:**
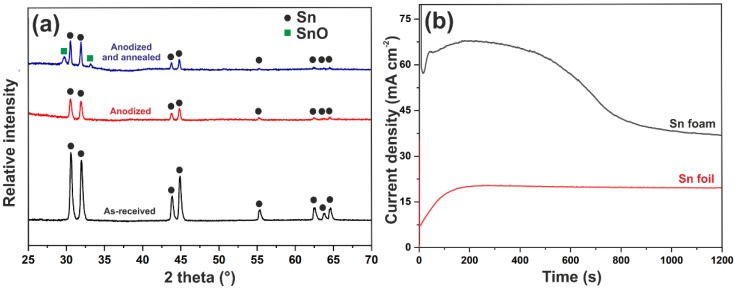
(**a**) X-ray diffraction (XRD) patterns of the as-received Sn foam (black line), Sn foam after 20 min of anodization (red line), and Sn-foam after anodization and annealing in air at 200 °C for 2 h (blue line); (**b**) current density vs. time curves recorded during the anodization process conducted on the Sn foil (red line) and Sn foam (black line) in 1 M NaOH at the potential of 4 V.

FE-SEM images of the metallic tin foam obtained by electrodeposition for 60 s in acidulated 20 mM SnCl_2_·2H_2_O at the potential of 6 V are shown in Figure 1. It can be seen that the porous foam consists of randomly distributed dendrites grown uniformly on the entire metal surface (see also the optical microscope image in Figure S2H in the Supplementary Information). The estimated average value of pore diameter near the surface is ~35 µm. However, a closer inspection reveals that the voids with a diameter of ~15 µm appeared in a deeper part of the deposit. This is in excellent agreement with the results obtained by other authors showing that the pore size of the foam increases with the distance from the substrate due to the coalescence of hydrogen bubbles [9]. A typical individual dendrite consists of a stem with a diameter of ~1.5 µm and side branches with a thickness of ~200 nm (see Figure 1c). The XRD pattern of electrodeposited foam shown in Figure 2a confirms that tetragonal Sn (ICDD card no. 00-004-673) is the main component of the obtained foam.

As-obtained Sn foams were superhydrophilic with a CA < 10° (the image of water droplet just after the contact with the surface is shown as an inset in Figure 1a). Completely opposite wetting behavior of Sn foams was observed by Cao et al. [37], who found them either hydrophobic or even superhydrophobic (with the CA range of 120–165°), depending on electrodeposition conditions. The authors attributed this phenomenon mainly to the self-passivation of the Sn surface resulting in Sn/SnO_x_ foams. However, since in both cases, Sn foams were stored at ambient conditions enabling the self-passivation of the electrodeposit, the opposite wetting behavior of as-received tin foams must be explained in terms of their different morphology caused by different electrodeposition conditions (mainly applied potential and concentration of Sn^2+^ ions). Indeed, layers obtained by Cao et al. are composed of more uniform dendrites with a stem and branches having a quite similar size, and an average pore diameter near the surface which is much larger (>100 µm) than in our case (see above) [37]. Taking the above into consideration, it can be stated that the self-passivation of Sn foam is not always enough to achieve surface superhydrophobicity.

Since an oxide film can be generated on the Sn surface by its annealing in air at 200 °C, we decided to check if such a thermal treatment will affect the wettability of Sn foams. As can be seen in Figure 1d, after annealing the foam became superhydrophobic with the average CA of 150 ± 4°. Moreover, low adhesion between the surface and the water droplet was observed, since the droplet could hardly be transferred from the syringe tip to the surface. This indicates that the water at the thermally passivated Sn/SnO_x_ foam was in the Cassie–Baxter state, i.e., the droplet is not able to effectively wet the microstructure due to the air trapped underneath. 

Such a dramatic change in wettability caused by annealing could be attributed to two factors. Firstly, a thin layer of oxide is formed on the metal surface during thermal treatment. Secondly, some changes in the morphology occurred during annealing, as can be seen in Figure 1d–f. It should be mentioned that the annealing temperature of 200 °C is only slightly lower from the melting point of bulk tin (~230 °C) therefore, some metal softening leading to changes in the morphology of dendrites can be expected. It is clear that after annealing, the foam structure became denser, and individual branches within the dendrites are melted or bonded together by thermally generated oxide (especially visible in Figure 1e,f). However, taking into account that in case of the plain Sn foil, a thermally generated oxide layer significantly increases hydrophilicity of the surface (CA for the bare Sn foil is 88 ± 1° and decreases to 57 ± 2° after annealing—see Figure S3 in the Supplementary Information), the morphological changes occurring during thermal treatment are mainly responsible for the superhydrophobic nature of annealed Sn foams.

As prepared Sn foams were then used as starting materials for anodization in order to verify if the formation of nanoporous oxide layer without damage of the foam structure is possible, and if so, what is the effect of such kind of the porous shell on properties of Sn/SnO_x_ foams.

### 3.2. Anodic Oxidation of Sn Foams

After electrodeposition, Sn foams were subjected to potentiostatic anodic oxidation in the alkaline electrolyte. All anodization conditions were developed in our previous works [24,32]; however, due to the micro/nanostructured character of foams, the process duration needs to be carefully reconsidered. In order to investigate this parameter, a series of anodizations were carried out with three different anodizing times ranging from 10 to 30 min. Anodization of the plain Sn foil was also performed for comparison. Figure 2b shows the current density vs. time plots recorded during anodic oxidation of the tin foam (black line) and foil (red line). Significant differences between processes carried out using the Sn foil and foam can be easily recognized. Firstly, in the case of foam anodization, the current values are significantly higher, which indicates a much higher working surface area of metal foams in comparison to the plain surface (as in both cases current densities were calculated with respect to the plain surface of the electrode). Secondly, the curve recorded during anodization of the Sn foam exhibits a significantly different and unusual shape. For both types of substrates, a current density drop caused by the formation of the compact passive oxide layer is observed immediately after applying the potential and it is followed by the current rise, being an indication of the pore formation (for detailed discussion of current density shapes and particular stages of anodization, please refer to our previous works [24,32,44,45]). After about 100 s of anodization of the Sn foil, a steady-state current is reached, and no significant changes are noticeable until the end of the process. Contrary to this, when the Sn foam was anodically oxidized, current stabilization is reached much faster (after c.a. 50 s of anodization) but after c.a. 500 s it starts to drop significantly. This phenomenon can be attributed to the complete anodization of thinnest parts of Sn dendrites within the foam. After about 1000 s of anodization, the current density reached a stable value indicating that most of the dendrites were successfully oxidized, and there are no significant changes in the active surface of the metallic Sn substrate on which oxide formation can occur. 

**Figure 3:**
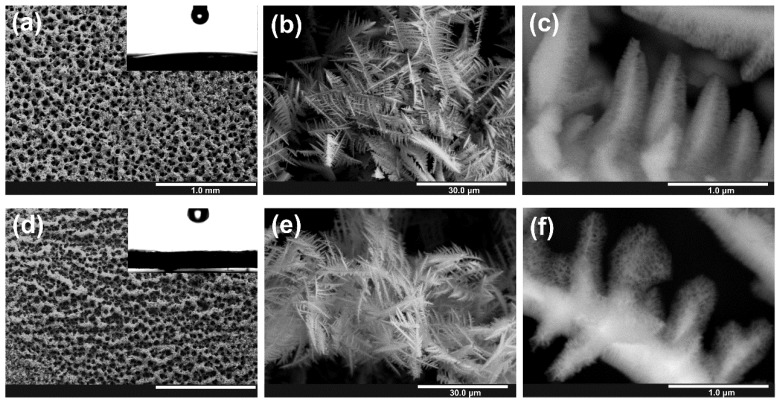
FE-SEM images of anodic Sn/SnO_x_ foams obtained by anodic oxidation of metallic foams in 1 M NaOH electrolyte at the potential of 4 V during 600 s (**a**–**c**) and 1200 s (**d**–**f**).

The FE-SEM images of as obtained nanostructured layers are presented in Figure 3. It can be seen that for samples anodized for 600 and 1200 s, the morphology of metal foam did not change significantly, and an open-porous structure is still preserved; likewise, a porous anodic oxide layer uniformly covers the whole metal surface. On the contrary, when the process is conducted for 1800 s, most of the foam delaminates from the substrate (see Figure S4 in the Supplementary Information) due to a significant drop in mechanical resistance of the material caused by the complete anodization of metallic dendrites. It is visible in Figure S4B that dendrites are crumbled and distorted. Therefore, only samples anodized for 600 and 1200 s were taken for further studies.

In all cases, the average diameter of nanochannels within the anodic film was estimated to be ~60 nm what is in accordance with values previously observed for anodic SnO_x_ formed on the Sn foil (for details see our previous works [24,44]). As can be seen in Figure 2a, no significant changes in XRD patterns of Sn foams were caused by anodization that indicates the amorphous or poorly crystalline nature of the anodic film and is consistent with our previous findings [44]. 

**Figure 4:**
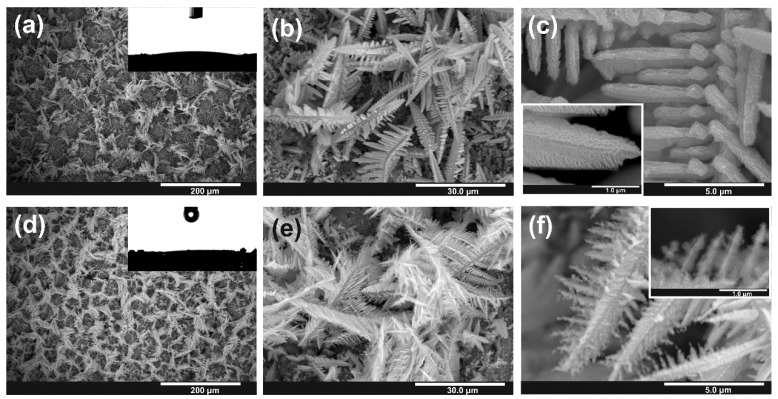
FE-SEM images of anodic Sn/SnOx anodized at 4 V for 600 s (**a**–**c**), and 1200 s (**d**–**f**) after annealing in air at 200 °C for 2 h.

The wettability of anodized samples was also studied. As we recently proved, the generation of nanoporous SnO_x_ film on a flat Sn foil significantly improves hydrophilicity of the surface [40]. As shown in insets in Figure 4a,d, the presence of porous oxide did not change the wettability of Sn foams, i.e., even after anodic oxidation, such layers remain superhydrophilic (CA < 10°). 

The as-obtained Sn/SnO_x_ foams were then subjected to annealing in air at 200 °C and SEM images of obtained materials are collected in Figure 4. Contrary to the annealed “bare” Sn foam, no significant changes in the microstructure of the foam were caused by thermal treatment. Dendrites are still preserved, and their branches remain separated (see especially Figure 4c).

The XRD pattern of annealed sample exhibits, except apparent diffraction peaks corresponding to metallic tin, two additional low-intensity peaks located at c.a. 30° and 33°, which give the evidence for the presence of the SnO crystalline phase after thermal treatment. This can be, on the one hand, the result of thermal oxidation of the remaining Sn (especially significant in case of micro/nanostructured substrate), and on the other hand, the crystallization of Sn^2+^-rich domains within the as-anodized non-stoichiometric anodic SnO_x_ structure [29,40].

Moreover, for the samples anodized for 1200 s, a noticeable degradation of oxidized dendrites occurs during annealing resulting in a gradual disintegration of the thinnest parts of branches (see Figure 4f). Therefore, at this point, it can be stated that in order to maintain integrity of dendrites, careful optimization of the anodizing duration together with conditions applied during thermal treatment (if needed for a particular application) is strongly required.

What is interesting, contrary to the “bare” Sn foam, those anodically oxidized remain superhydrophilic even after annealing (see insets in Figure 4a,d). It means that the presence of a nanoporous SnO_x_ shell efficiently suppresses significant morphological changes during thermal treatment.

### 3.3. Photoelectrochemical Tests of Sn/SnO_x_ Foams

**Figure 5:**
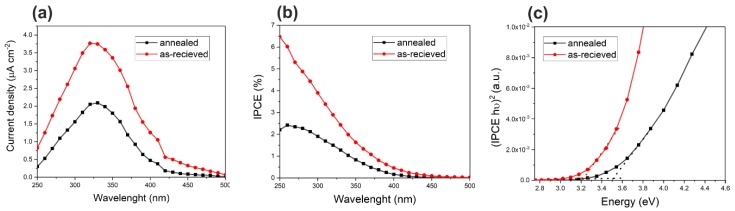
Photocurrent (**a**) and incident photon to current efficiency (IPCE) (**b**) as a function of the incident wavelength together with (IPCE hν)^2^ vs. (hν) plot (**c**) for anodic layers obtained on metal foams.

Anodized Sn foams were also subjected to some preliminary photoelectrochemical measurements. Unfortunately, the electrochemical stability of samples anodized for 10 min was insufficient to perform a full set of measurements. Therefore, only Sn/SnO_x_ systems obtained after 20 min of anodic oxidation were tested. An example of the chronoamperometric curve recorded during the sequential illumination of as-synthesized Sn/SnO_x_ is presented in Figure S5 (see the Supplementary Information). Although noticeable dark currents appeared during anodic polarization of the sample, they were stable enough to allow recording of the photocurrent spectrum, which is shown in Figure 5a (red line).

It has been proven, that annealing of anodic SnO_x_ layers formed on the plain Sn foil not only can improve the photoresponse of anode [29,40,44] resulting in both higher photocurrents and enhanced electrode stability, but also can affect the Sn^2+^ content and, in consequence, bandgap of the semiconductor [40]. For this reason, the photoresponse of annealed Sn/SnO_x_ foams was also tested, and the obtained photocurrent spectrum is also shown in Figure 5a (black line).

Incident photon to current efficiency (IPCE) values was calculated for both studied samples using Equation (1),

(1) $$IPCE=1240\cdot \frac{I_{p}\left(\lambda \right)}{P\left(\lambda \right)\cdot \lambda }$$

where: I_p_ (λ) is photocurrent density (A·m^−2^), P(λ) is the incident power density of light (W·m^−2^) and λ is the wavelength (nm), and the resulting IPCE spectra are shown in Figure 5b.

Surprisingly, contrary to the previous works [29,40], the higher photoelectrochemical response was observed for the as-synthesized sample. A significant IPCE drop caused by annealing can be attributed to the different behavior of micro/nanostructured Sn during thermal treatment, including further oxidation of the metallic substrate resulting in the generation of compact SnO layer at the Sn/SnO_x_ interface. However the most important is the partial disintegration and fragmentation of the thinnest parts of dendrites (see Figure 4f) resulting in a lower active surface area of the photoanode and worse electrical contact between the semiconductor and metallic current collector.

Finally, the bandgap for both as-synthesized and annealed Sn/SnO_x_ foams was estimated from (IPCE hυ)^2^ vs. (hυ) plots (Tauc plots) presented in Figure 5c. Again, contrary to anodic SnO_x_ obtained directly on Sn foils, the bandgap for the annealed sample was found to be significantly higher (3.51 eV) than for as-synthesized one (3.21 eV). 

However, it should be emphasized that the results described above should be treated only as preliminary evidence that such kind of hierarchical Sn/SnO_x_ systems can exhibit significant photoelectrochemical activity. However, further extensive studies should be performed in order to find optimal procedures for both the synthesis of the material itself and its post-treatment. Particular emphasis should be put on improving the conversion efficiency, but mostly, the stability of the material. Such works are currently being carried out in our group.

## 4. Conclusions

In summary, it has been proven for the first time that hierarchical nanoporous tin/tin oxide systems can be successfully obtained by electrochemical deposition of the metallic Sn foam in an acidic electrolyte followed by its anodic oxidation in NaOH. Moreover, careful optimization of the anodizing duration allows the formation of nanoporous oxide shell on the metallic core without significant changes in the foam morphology. Contrary to thermally oxidized foams, which are hydrophobic, nanoporous tin/tin oxides were found to be superhydrophilic even after further thermal treatment that means that anodic oxidation gives the opportunity to tune the wetting behavior of this kind of porous materials. It is strongly expected that such hierarchical nanostructured composites can be very promising candidates for various applications, including energy conversion and storage systems. Finally, it is believed that similar procedures of electrochemical deposition and anodization can also be employed for the fabrication of similar hierarchical metal/metal oxide nanostructures of other elements.

## Supplementary Materials

The following are available online at https://www.mdpi.com/2079-4991/10/3/410/s1, Figure S1: Current density vs. time curve recorded during Sn electrodeposition in the electrolyte containing 20 mM SnCl_2_·2H_2_O and 1.5 M H_2_SO_4_ at the potential of 6 V, Figure S2: Optical microscope images of Sn samples with various pre-treatments (no pre-treatment—A; roughed by sandpaper—C; activated in HCl—E; roughed and activated—G) together with Sn foams electrodeposited on particular substrates (B, D, F, H), Figure S3: Optical images of water droplets on the surface of plain Sn foil before (A) and after (B) annealing in air at 200 °C for 2 h, Figure S4: FE-SEM images of Sn foams after 30 min of anodization in 1 M NaOH at 4 V. Low magnification (A), and higher magnification (B) images, Figure S5: Chronoamperometric curve recorded during the sequential illumination of the Sn/SnO_x_ foam with the light of different wavelengths (with a step of 10 nm) at the potential of 0.9 V vs. SCE.
