# Supplementary material for: Hierarchical Nanoporous Sn/SnOx Systems Obtained by Anodic Oxidation of Electrochemically Deposited Sn Nanofoams
Source: Nanomaterials (Basel). 2020 Feb 26;10(3):410. doi: 10.3390/nano10030410 (PMC7152847; doi:10.3390/nano10030410)
Supplement: Supplementary file 1 [file nanomaterials-10-00410-s001.pdf]

Supplementary Information

Hierarchical nanoporous Sn/SnO_x systems obtained by anodic oxidation of electrochemically deposited Sn nanofoams

Magdalena Gurgul, Anton S. Lytvynenko, Magdalena Jarosz, Karolina Gawlak,
Grzegorz D. Sulka, Leszek Zaraska

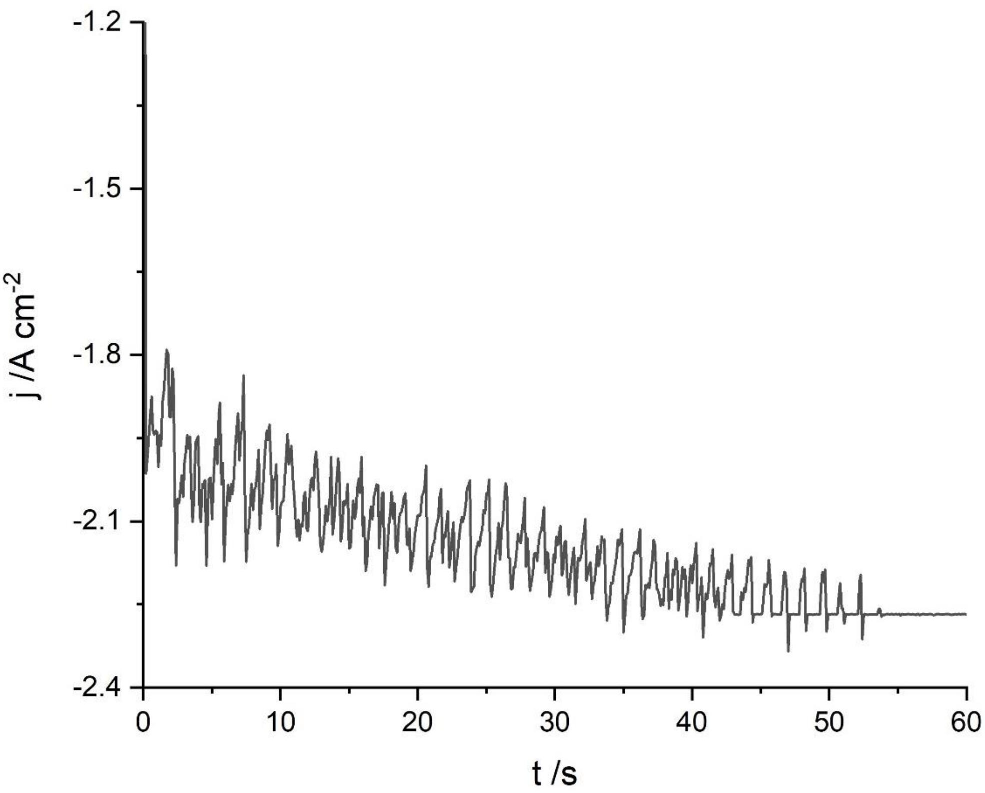

Figure S1. Current density vs. time curve recorded during Sn electrodeposition in the electrolyte containing 20 mM $SnCl_2 \cdot 2H_2O$ and 1.5 M H_2SO_4 at the potential of 6 V.

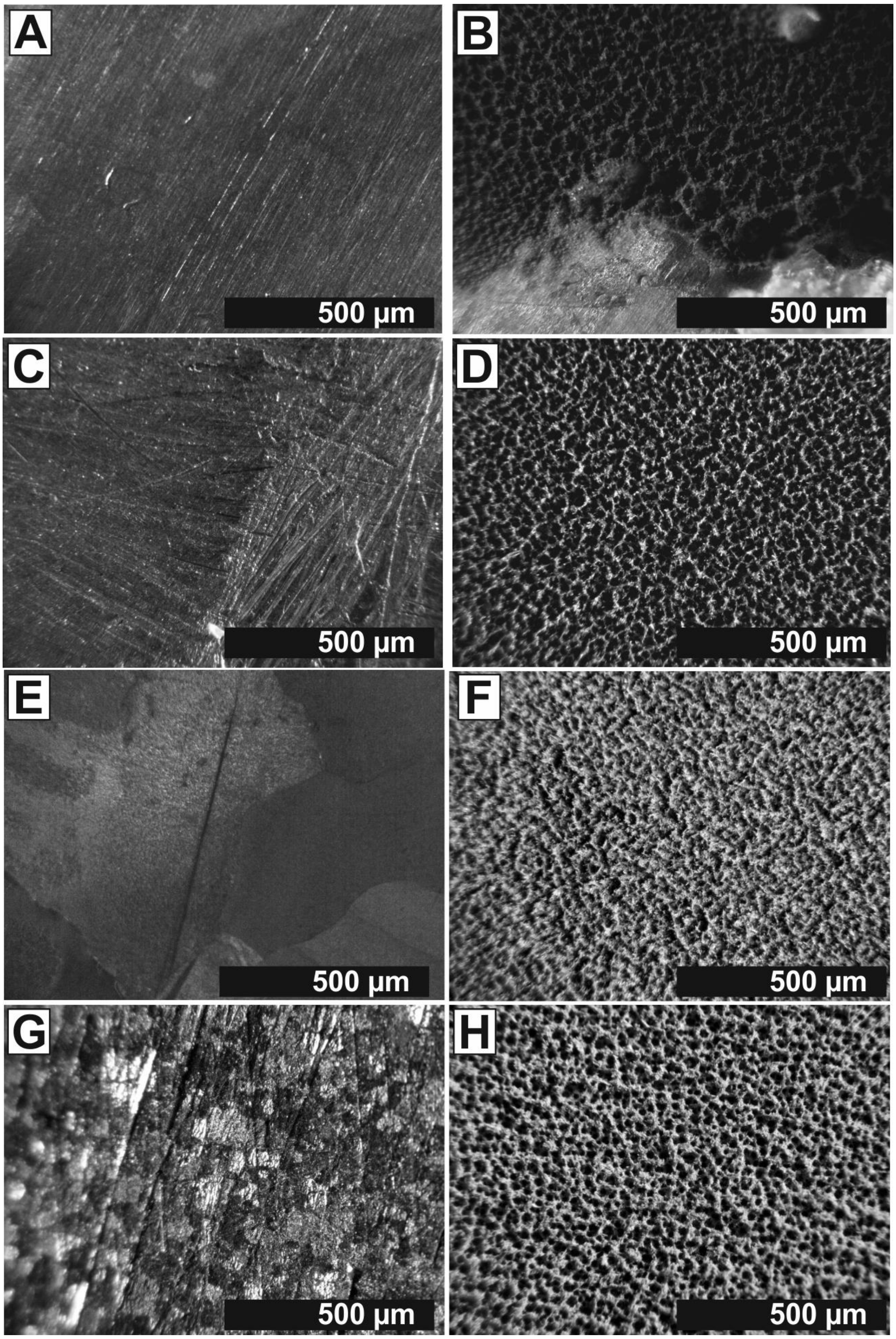

Figure S2. Optical microscope images of Sn samples with various pre-treatments (no pre-treatment – A; roughed by sandpaper – C; activated in HCl – E; roughed and activated – G) together with Sn foams electrodeposited on particular substrates (B, D, F, H).

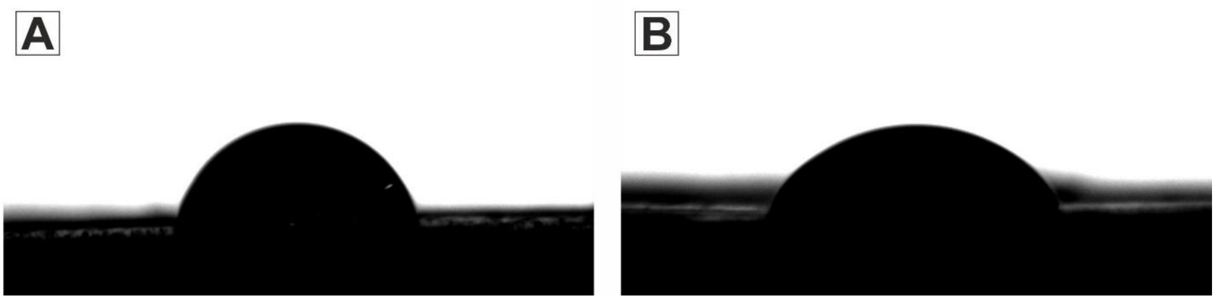

Figure S3. Optical images of water droplets on the surface of plain Sn foil before (A) and after (B) annealing in air at 200 °C for 2 h.

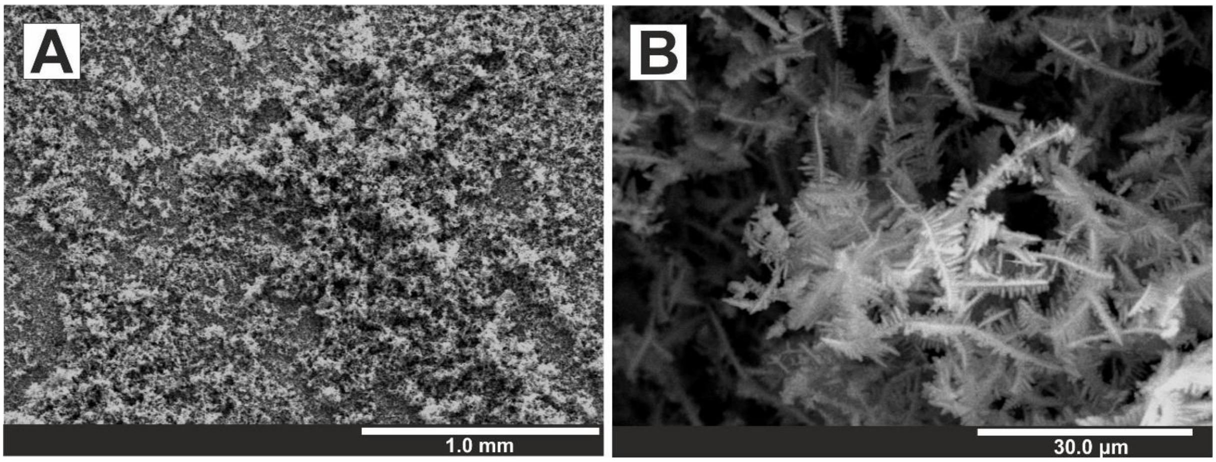

Figure S4. FE-SEM images of Sn foams after 30 min of anodization in 1 M NaOH at 4 V. Low magnification (A), and higher magnification (B) images.

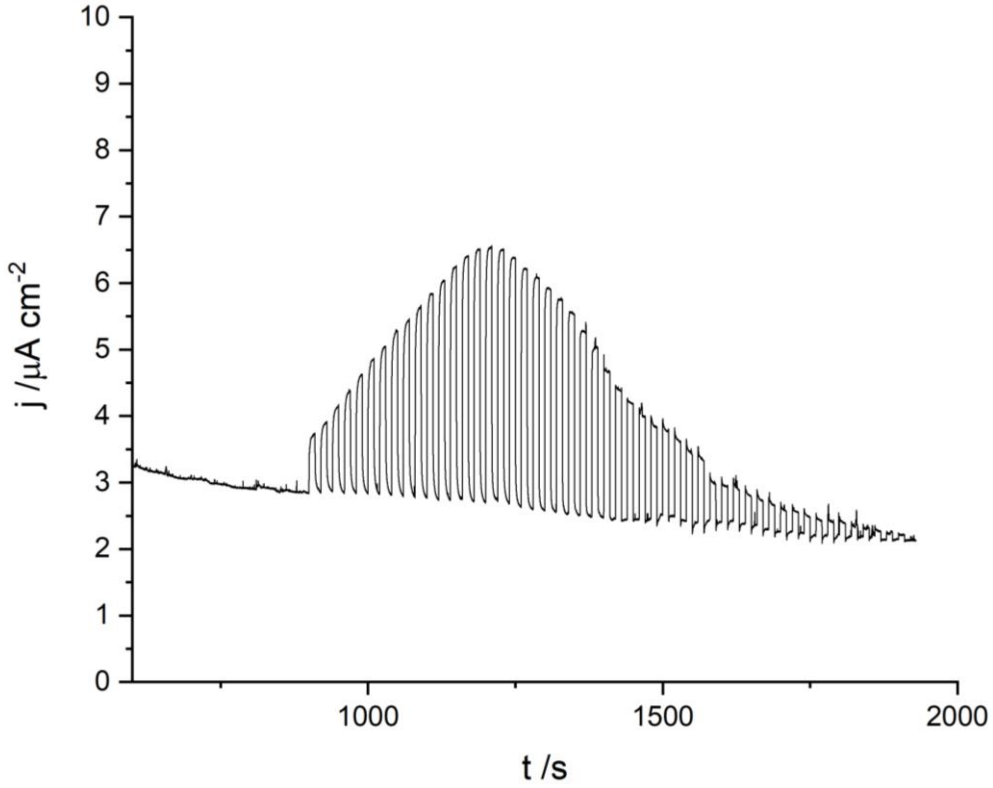

Figure S5. Chronoamperometric curve recorded during the sequential illumination of the Sn/SnO_x foam with the light of different wavelengths (with a step of 10 nm) at the potential of 0.9 V vs. SCE.
